# Review of the western African millipede genus *Diaphorodesmus* Silvestri, 1896 (Diplopoda, Polydesmida, Chelodesmidae), with the description of a similar, but new monotypic genus from Cameroon

**DOI:** 10.3897/zookeys.600.9345

**Published:** 2016-06-22

**Authors:** Didier VandenSpiegel, Sergei I. Golovatch, Jean-Paul Mauriès

**Affiliations:** 1 Musée Royal de l’Afrique Centrale, B-3480 Tervuren, Belgium; 2 Institute for Problems of Ecology and Evolution, Russian Academy of Sciences, Leninsky pr. 33, Moscow 119071, Russia; 3 Muséum national d’Histoire naturelle, Département Systématique & Evolution, CP n°53, 61 rue Buffon, 75005 Paris, France

**Keywords:** Taxonomy, synonymy, new species, Cameroon, Nigeria, Equatorial Guinea

## Abstract

The genus *Diaphorodesmus* is revised and shown to comprise only a single species, *Diaphorodesmus dorsicornis* (Porat, 1894) by priority, with the only other formal congener, *Diaphorodesmus attemsii* Verhoeff, 1938, considered as its junior subjective synonym, **syn. n.** A new monotypic genus, *Diaphorodesmoides* **gen. n.**, is created to include *Diaphorodesmoides lamottei* **sp. n.**, from southwestern Cameroon. Both these genera seem to be especially similar in sharing remarkable dorsal horns on metaterga 2–4, a unique synapomorphy in the basically Afrotropical subfamily Prepodesminae, family Chelodesmidae, to which they belong. In contrast to *Diaphorodesmus* which shows two, increasingly short, paramedian horns on each of metaterga 2–4, the ozopores borne on distinct porosteles, and the gonopod prefemoral process and solenophore less strongly elaborate, *Diaphorodesmoides* **gen. n.** has a single, increasingly large, central horn on each of metaterga 2–4, the ozopores opening flush dorsolaterally on the surface of poriferous paraterga, and both the gonopod prefemoral process and solenophore especially complex. The genus *Campodesmoides* VandenSpiegel, Golovatch & Nzoko Fiemapong, 2015, and its sole, and type, species *Campodesmoides corniger* VandenSpiegel, Golovatch & Nzoko Fiemapong, 2015, are transferred from Campodesmidae to Chelodesmidae and formally synonymized with *Diaphorodesmus* and *Diaphorodesmus dorsicornis*, both **syn. n.**

## Introduction

The western African genus *Diaphorodesmus* Silvestri, 1896, was erected by Silvestri (1896) to encompass a single species that Porat (1894) had described as *Paradesmus dorsicornis* Porat, 1894, from Cameroon. The original description and most of the illustrations as presented by Porat (1894) were quite adequate for that time, showing almost all necessary details of body structure, including the remarkably strong, suberect, paramedian horns gradually decreasing in size on metaterga 2–4. The syntypes were said to be abundant, mostly taken at N’dian and Kitta. Only the gonopod was depicted too small and schematically, apparently this being one of the reasons for subsequent confusion.

Carl (1905), based on material from Cabo San Juan, then Spanish Guinea, now Equatorial Guinea, and, later, Attems (1931, 1938), based on rich samples coming from Mukonje Farm, Bibundi and Victoria, Cameroon, provided detailed descriptions and very clear illustrations of what they identified as *Diaphorodesmus dorsicornis*.

Verhoeff (1938), having studied some more material of *Diaphorodesmus* from Cameroon, yet with neither the number of specimens nor any precise locality indicated, came to the conclusion that what Attems (1931) had taken for *Diaphorodesmus dorsicornis* was actually a different species he named *Diaphorodesmus attemsii* Verhoeff, 1938. In addition, he illustrated the gonopod of what he believed to be *Diaphorodesmus dorsicornis* and, in a tabular form, also listed the main differences in body structure between the two species, as follows (translated from German).

**Table:** 

*Diaphorodesmus dorsicornis* Porat, Verh. The three pairs of dorsal processes on diplosomites 2–4 are similarly well-developed; that of the 4^th^ not displaced from the posterior edge. 4^th^ metatergite with 6 acute anterior tubercles, the two paramedian the largest.	*Diaphorodesmus attemsii* Verh. Of the three rows of dorsal processes those on the 4^th^ segment are not only smaller than the others, but also completely removed from the posterior edge. 4^th^ metatergite with 4 projections, all about the same size.

Besides this, Verhoeff (1938) created the subfamily Odontokrepinae (recte: Odontokrepidinae) Verhoeff, 1938, to harbour only two genera: *Diaphorodesmus* and *Odontokrepis* Attems, 1898. The latter genus was said to be distinguished from the former by the presence of tergal horns on segments 2–4, and of porosteles. Attems (1940) regarded *Odontokrepis* a dubious genus with three species from Cameroon, whereas Hoffman (1980) treated it as a junior synonym of *Anisodesmus* Cook, 1895, with three species from Liberia (!), and the subfamily Odontokrepidinae as a junior synonym of Prepodesminae Cook, 1896.

Hoffman evidently believed that Verhoeff (1938) had erred as well in regarding his sample as representing a true *Diaphorodesmus dorsicornis*. He drew the gonopod of a syntype of *Diaphorodesmus dorsicornis*, still kept in the Porat collection at the Naturhistoriska Riksmuseet in Stockholm (NHRS), Sweden (Fig. 3A, B), and the gonopod of a ♂ from Victoria, Cameroon (housed in the Naturhistorisches Museum Wien (NHMW), Vienna, Austria) which Attems (1931, 1938) had identified as *Diaphorodesmus dorsicornis* and which Verhoeff (1938) had assigned to *Diaphorodesmus attemsii* (Fig. 3C, D). Hoffman also abundantly illustrated (Fig. 2) a ♂ from Port Harcourt, Rivers State, Nigeria (likely still housed in the Virginia Museum of Natural History where Hoffman worked), and assigned it to the species that Verhoeff (1938) had considered as a true *Diaphorodesmus dorsicornis*. Although Verhoeff’s (1938) sample from an unknown place in Cameroon was different from the ♂ from Port Harcourt, Hoffman provisionally referred both to a new species. As a result, Hoffman (1980), in the only published account of *Diaphorodesmus*, said that the genus contained three species from Cameroon.

The present paper has largely been prompted by the recent description of *Campodesmoides* VandenSpiegel, Golovatch & Nzoko Fiemapong, 2015, a monobasic genus that only encompasses the type-species, *Campodesmoides corniger* VandenSpiegel, Golovatch & Nzoko Fiemapong, 2015, from Cameroon (VandenSpiegel et al. 2015). That genus was erroneously assigned to the endemic western African family Campodesmidae, but in fact both the genus and species are junior synonyms of *Diaphorodesmus* and *Diaphorodesmus dorsicornis*, respectively, in the basically Afrotropical subfamily Prepodesminae Cook, 1896, family Chelodesmidae Cook, 1895.

To correct the mistake, we have been able to retrieve the unpublished relevant archives of the late R.L. Hoffman, housed in the Virginia Museum of Natural History, Martinsville, Virginia, U.S.A. In addition, we have gathered all relevant information concerning the type series of *Diaphorodesmus attemsii*, kept at the NHMW. This, plus several, largely unpublished samples received for study from the collections of the Muséum national d'Histoire naturelle (MNHN), Paris, France, the Natural History Museum of Denmark (ZMUC), Copenhagen, Denmark, and the Bayerische Zoologische Staatssammlung
(ZSM), Munich, Germany, has allowed us not only to finally clarify the tangled history of studies on *Diaphorodesmus*, but also to add a new genus and species described below.

## Material and methods

The material treated here derives from the collections of the Musée Royal de l'Afrique Centrale (MRAC), Tervuren, Belgium, the MNHN, the ZMUC, and the ZSM. The samples are stored in 70% ethanol. Specimens for scanning electron microscopy (SEM) were air-dried, mounted on aluminium stubs, coated with gold and studied using a JEOL JSM-6480LV scanning electron microscope. Photographs were taken with a Leica DFC 500 digital camera mounted on a Leica MZ16A stereo microscope. Images were processed with Leica Application Suite software.

In the species catalogue section, D stands for a description or descriptive notes (sometimes also including a key, discussion, new status, synonymy or combination), and R for new or old records.

## Results

### Class Diplopoda Blainville-Gervais, 1844 Order Polydesmida Leach, 1814 Family Chelodesmidae Cook, 1895

#### Genus Diaphorodesmus

Taxon classificationAnimaliaPolydesmidaChelodesmidae

Silvestri, 1896

Diaphorodesmus Silvestri, 1896: 197.
Diaphorodesmus – Cook 1896: 16; Attems 1899: 311; 1931: 91; 1938: 409; Carl 1905: 271; Verhoeff 1938: 166; Hoffman 1980: 155.
Campodesmoides VandenSpiegel, Golovatch & Nzoko Fiemapong, 2015, **syn. n.**
Campodesmoides corniger VandenSpiegel, Golovatch & Nzoko Fiemapong, 2015, by original designation. **Type species.**

##### Type species.

*Paradesmus dorsicornis* Porat, 1894, by original designation.

##### Diagnosis.

A genus of Prepodesminae, Chelodesmidae that is distinguished by the presence of conspicuous paramedian, increasingly short, dorsal, horns on metaterga 2–4, coupled with the normal pore formula: 5, 7, 9, 10, 12, 13, 15–19, the ozopores being borne on conspicuous porosteles; the spiracles are small and inconspicuous; and the gonopod telopodites suberect, *in situ* directed forward, held parallel to each other, not crossing mesally; prefemoral (= densely setose) part erect, taking up about 2/3 of total gonotelopodite length, without a femorite part, but with a prominent dorsal process (**pfp**), set off from acropodite by a distinct cingulum; acropodite clearly twisted, divided parabasally into one smaller dorsobasal lobule (**lo**) and two large lamellar lobes, the ventral lobe forming a solenophore (**sph**) to support a dorsal solenomere lobe (**slo**) with only an indistinct, small solenomere proper on top.

#### Diaphorodesmus dorsicornis

Taxon classificationAnimaliaPolydesmidaChelodesmidae

(Porat, 1894)

Figs 1 , 2 , 3 , 4 , 5 , 6 , 7 , 12

**Figure 1.**
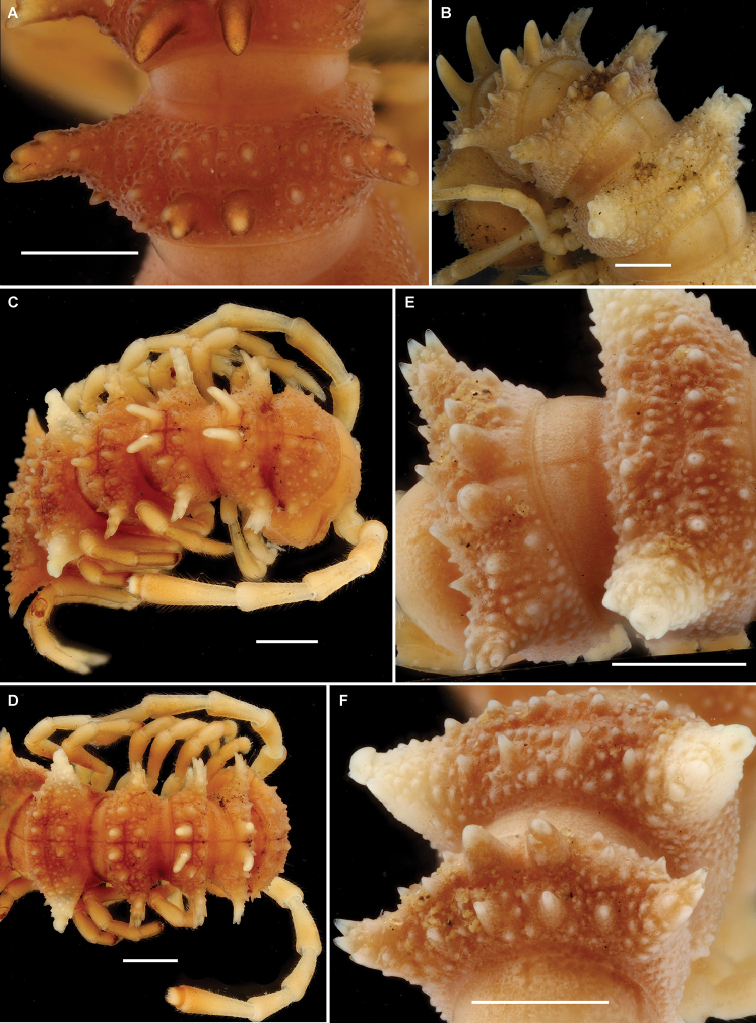
*Diaphorodesmus dorsicornis* (Porat, 1894). **A** Metatergum 4 of a ♂ (ZMUC) from Osomba/Calabar, Nigeria, dorsal view **B** Anterior body part of a ♂ (NHMW) from Bibundi, Cameroon, dorsolateral view **C, D** Anterior body part of a ♂ (MNHN) from Kumba, Cameroon, dorsolateral and dorsal views, respectively **E, F** Metaterga 4 and 5 of a ♂ (ZSM) from an unknown locality in Cameroon, dorsolateral (4^th^ to the left) and dorsal (4^th^ at the bottom) views, respectively. Photos by J. Brecko (**A**, **C–F**) and N. Akkari (**B**).

Paradesmus dorsicornis Porat, 1894: 33, figs 3–3c (D).
Diaphorodesmus dorsicornis – Silvestri 1896: 197 (D) (erection and typification of Diaphorodesmus); Cook 1896: 16 (D); Attems 1899: 312, plate 7, fig. 167 (D) (reiterated original description and a reproduced original figure); 1931: 100, figs 147–151 (D, R); 1938: 409, figs 451–452 (D, R); Carl 1905: 271, plate 6, fig. 1–1a (D, R).
Diaphorodesmus attemsii Verhoeff, 1938: 167, figs 1–3 (D), **syn. n.**
Diaphorodesmus attemsii – Attems 1940: 560 (D, R).
Campodesmoides corniger VandenSpiegel, Golovatch & Nzoko Fiemapong, 2015: 2, figs 1–3 (D), **syn. n.**

##### Material examined.

Apart from the type series of *Campodesmoides corniger*, deposited at MRAC (VandenSpiegel et al. 2015), the following unpublished samples are available.

1 ♂ (MNHN JB254), Cameroon, Kumba, 25.XI.1975, leg. M. Lamotte (*Diaphorodesmus dorsicornis*, det. J.-P. Mauriès); 5 ♂, 2 ♀ (ZMUC), eastern Nigeria, Osomba 56 miles from Calabar, 17.VI.1965; 1 ♀ (ZMUC), eastern Nigeria, 1963, all leg. V. Schiøtz (*Diaphorodesmus attemsii*, all det. H. Enghoff).

##### Revised published material.

1 ♂, 2 juveniles (fragments of caudal body part only) (ZSM Reg. No. A 20052425 + slide A 20035316), “Kamerun”, without further information (*Diaphorodesmus dorsicornis*, det. K.W. Verhoeff).

##### Remarks.

**Figure 2.**
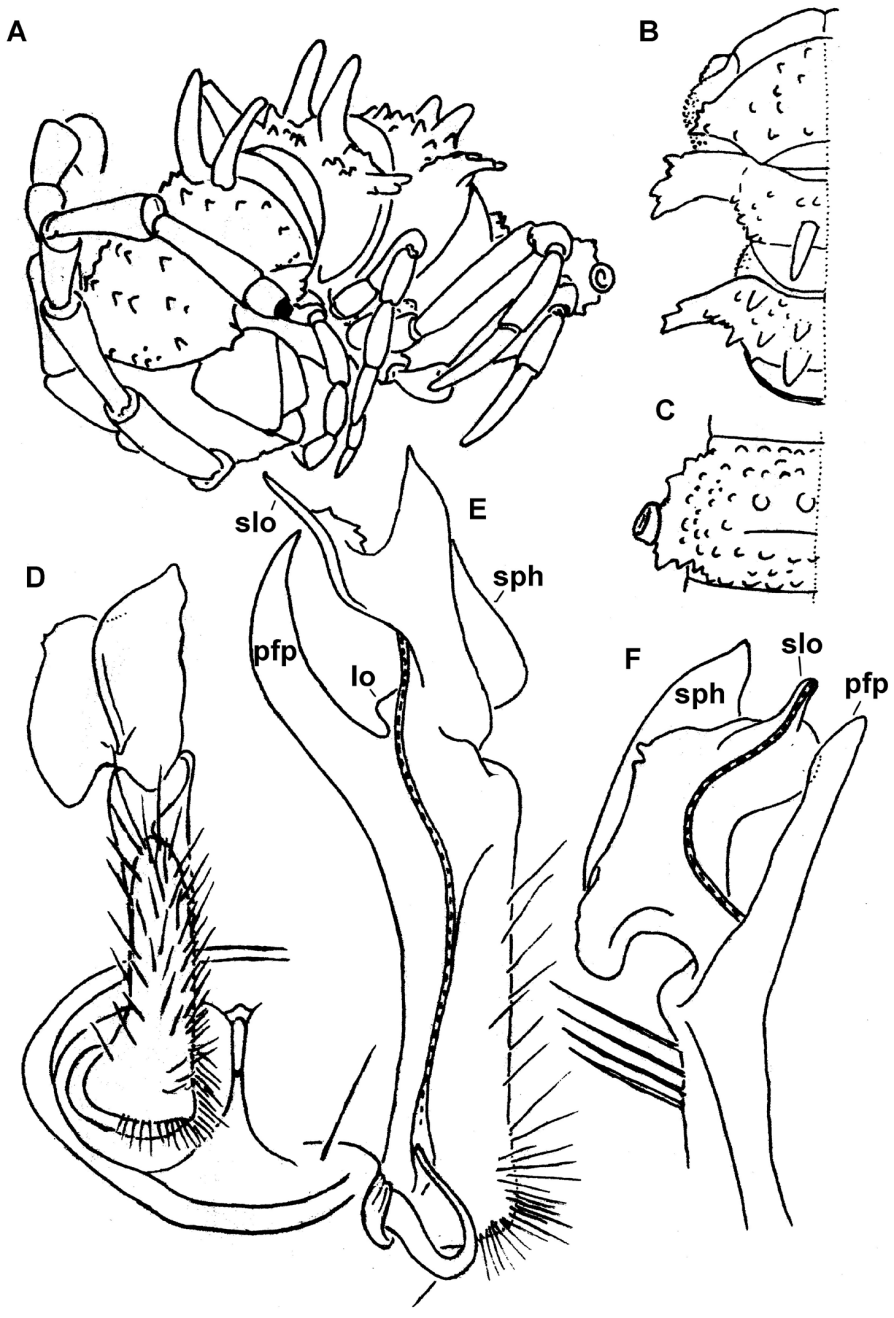
*Diaphorodesmus dorsicornis* (Porat, 1894), ♂ from Port Harcourt, Nigeria. **A, B** Anterior body part, sublateral and dorsal views, respectively. **C**. Metatergum 10, dorsal view. **D** Right gonopod *in situ*, ventral view **E, F** Left gonopod, mesal and lateral views, respectively. Del. R.L. Hoffman, drawn not to scale. Labels added by present authors; abbreviations explained in text.

**Figure 3.**
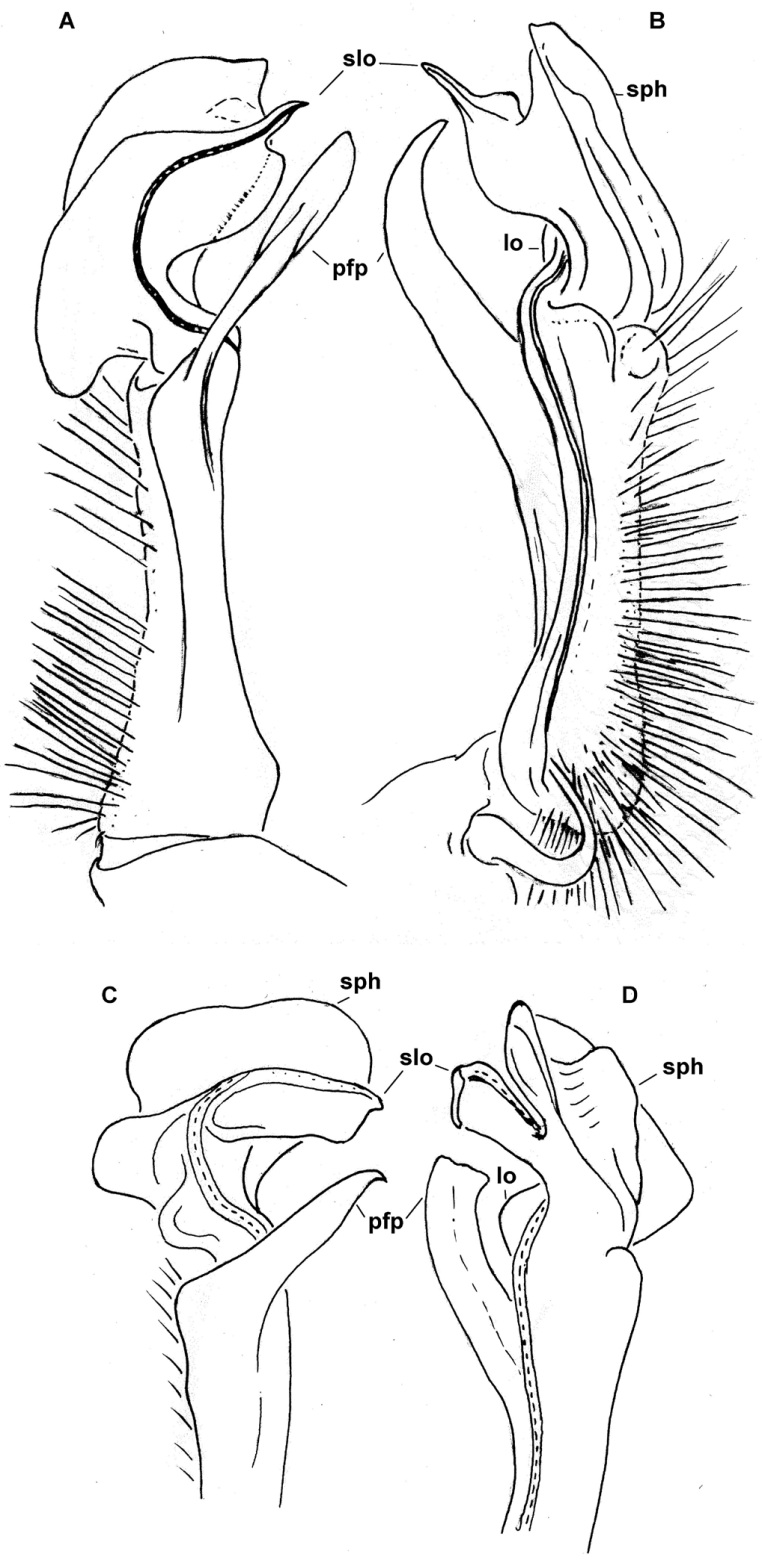
*Diaphorodesmus dorsicornis* (Porat, 1894). **A, B** Left gonopod of a ♂ syntype (NHRS) from an unspecified locality in Cameroon, lateral and mesal views, respectively **C, D** Distal part of the left gonopod of a syntype of “*Diaphorodesmus attemsii* Verhoeff, 1938” (Hamburg Museum?) from the Botanical Garden in Victoria, Cameroon, lateral and mesal views, respectively. Del. R.L. Hoffman, drawn not to scale. Labels added by present authors; abbreviations explained in text.

**Figure 4.**
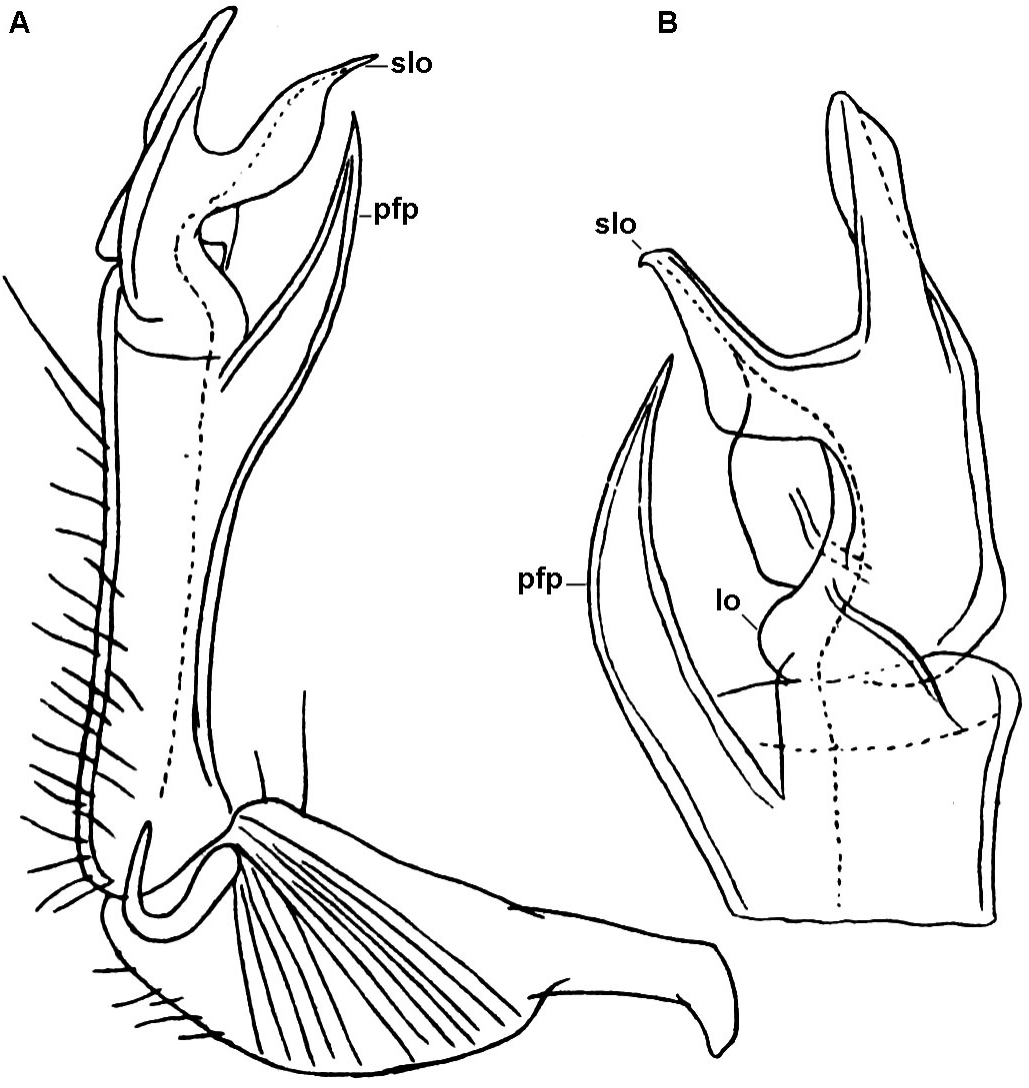
*Diaphorodesmus dorsicornis* (Porat, 1894). Gonopods of a ♂ (ZSM) from an unspecified locality in Cameroon. **A** Right gonopod, mesal view **B** Tip of left gonopod, mesal view. Del. K.W. Verhoeff, drawn not to scale. After Verhoeff (1938). Labels added by present authors; abbreviations explained in text.

**Figure 5.**
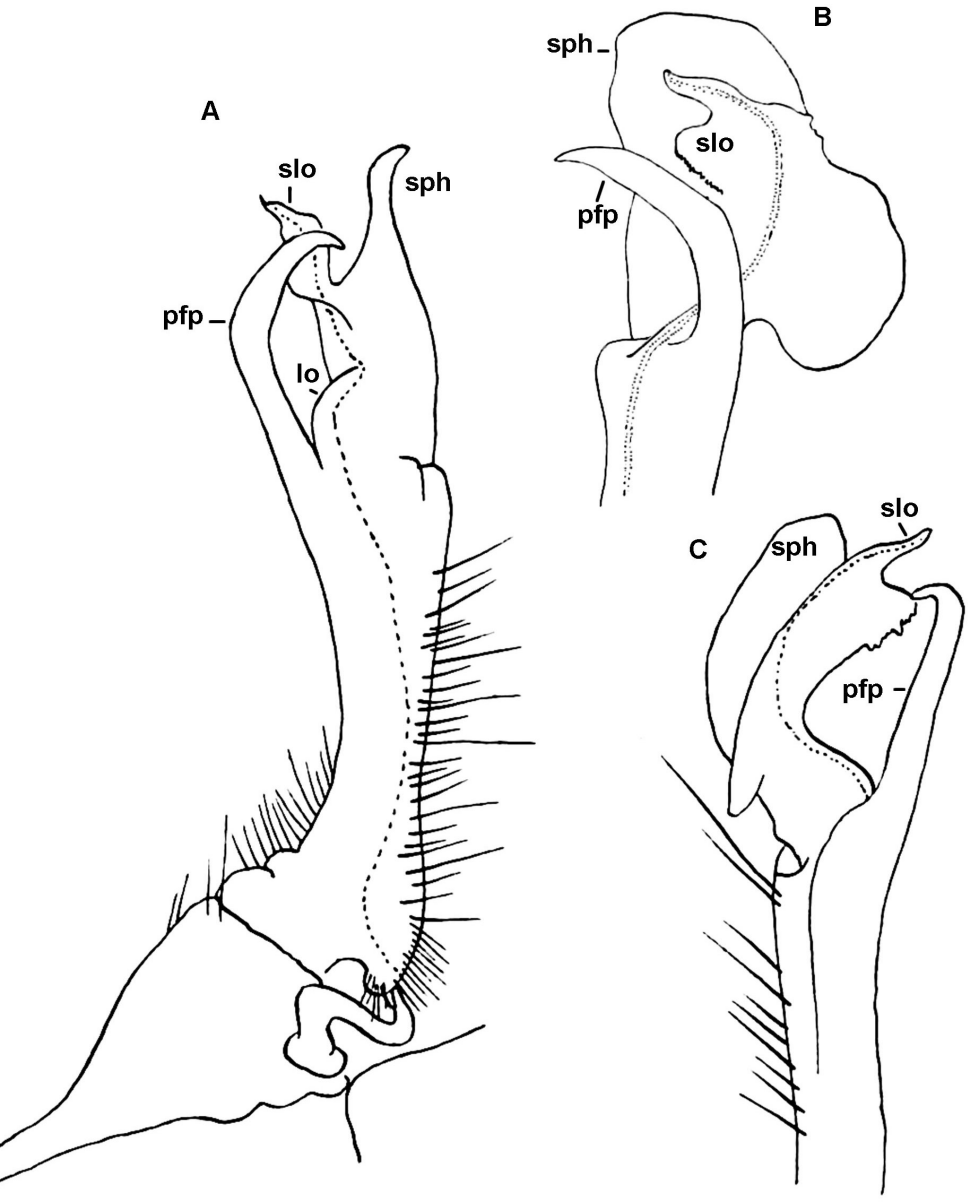
*Diaphorodesmus dorsicornis* (Porat, 1894). Gonopods of a ♂ syntype of “*Diaphorodesmus attemsii* Verhoeff, 1938” (NHMW) from an unspecified locality in Cameroon. **A** Left gonopod, mesal view **B** Tip of right gonopod, anterior view **C** Most of telopodite of right gonopod, lateral view. Del. C. Attems, drawn not to scale. After Attems (1931). Labels added by present authors; abbreviations explained in text.

**Figure 6.**
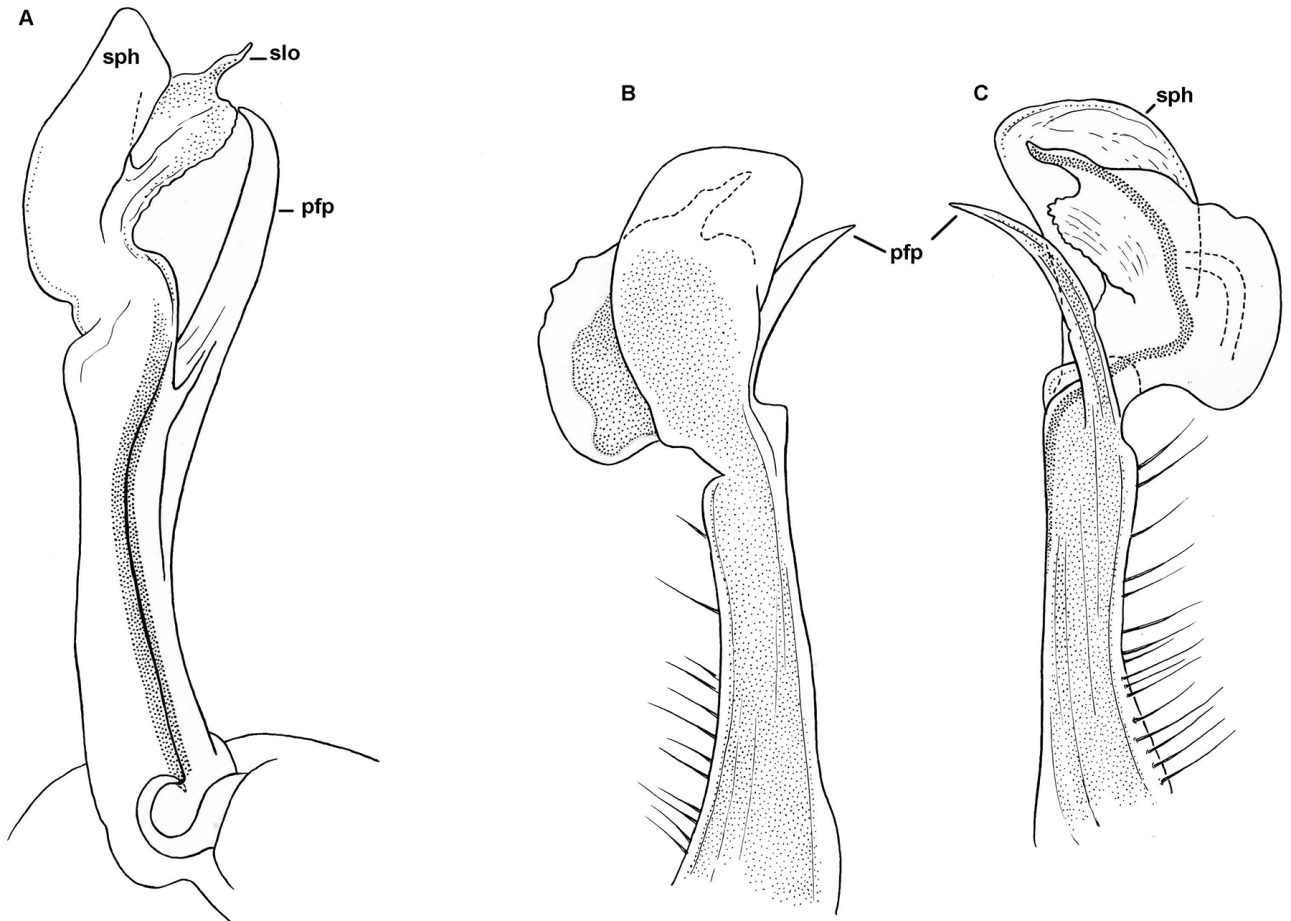
*Diaphorodesmus dorsicornis* (Porat, 1894). Gonopods of a ♂ (MNHN) from Kumba, Cameroon. **A** Right gonopod, mesal view **B–C** Telopodite of right gonopod, ventral and anterior views, respectively. Del. N. Bertoncini (MHNH). Labels added by present authors; abbreviations explained in text.

**Figure 7.**
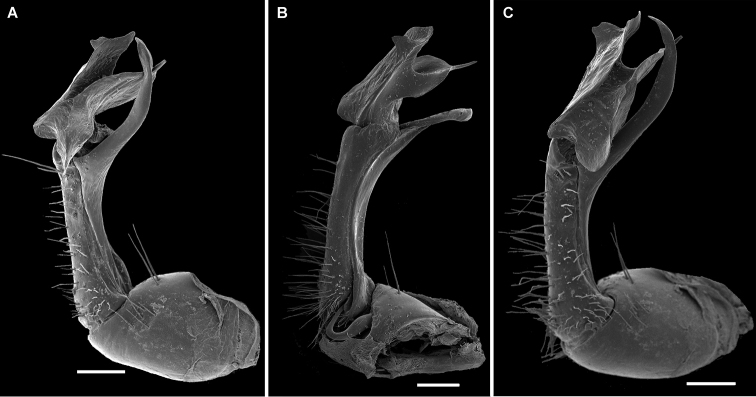
*Diaphorodesmus dorsicornis* (Porat, 1894). SEM micrographs of both gonopods of a ♂ of “*Diaphorodesmus attemsii* Verhoeff, 1938” (ZMUC) from Osomba/Calabar, Nigeria. **A, C** Left gonopod, lateral and sublateral views, respectively **C** Right gonopod, mesal view. Scale bars: 0.2 mm.

This species enjoys several descriptions, the latest of which (VandenSpiegel et al. 2015) is particularly complete and detailed. We only add here more pictures and drawings (Figs 1–7) to show evident variations in some somatic and gonopodal characters that bridge *Diaphorodesmus dorsicornis* and *Diaphorodesmus attemsii* and justify their synonymization.

Considering the measured material published elsewhere (Porat 1894; Attems 1938; VandenSpiegel et al. 2015) and here, body size variations are quite considerable both between individuals and, to a lesser degree, sexes: length 26–35 mm (♂, ♀), width of midbody pro- and metazonae 2.1–3.5 and 3.0–4.9 mm (♂) or 2.5–3.6 and 3.6–5.0 mm (♀), respectively. General coloration varies from yellow through grey-brown to blackish (Porat 1894; Carl 1905; Attems 1931, 1938; VandenSpiegel et al. 2015).

As regards the somatic characters mentioned by Verhoeff (1938) and quoted above that distinguish *Diaphorodesmus attemsii* from *Diaphorodesmus dorsicornis*, they are actually mistaken or reflecting individual variations. Thus, the dorsal horns on metaterga 4 are typically somewhat shorter in the ♀ compared to the ♂, and they tend to be more or less gradually and increasingly reduced from metatergum 2 to 4 in both sexes. The higher the horns on metatergum 4, the less strong their shift forward off the caudal margin. This shift is usually particularly apparent in the ♀.

The more or less evident cones in front of these horns are usually subequal in shape and size, 2+2, arranged in a transverse row (Fig. 1A, C, D). However, occasionally there are variations observed in shape and size of those cones as well. The pertinent material of Verhoeff (1938), at least the single adult ♂ at his disposal which is currently kept at the ZSM, shows the typical 2+2 (not 3+3!) cones, albeit the central pair is indeed a little larger than the lateral one, while the dorsal horns are relatively short, tuberculiform, clearly set off from the caudal margin of the metatergum (Fig, 1E, F). The gonopod structure of the ZSM ♂ is likewise closer to the one as depicted by Attems (1931) for “*Diaphorodesmus attemsii*” (Fig. 4).

The single relatively large sample in our hands, that from Osomba, shows the following variations in structure of metatergum 4. Most of the samples have rather long dorsal horns which often are even slightly curved caudad and set close to the caudal margin, with 2+2 subequal tubercles/cones in front. However, in one ♂ the situation is largely the same as described above for the ZSM ♂. It shows the gonopods typical of “*Diaphorodesmus attemsii*” as clearly depicted by Attems (1931, 1938) (Fig. 5) and used for SEM here (Fig. 7), both horns are shorter, rather tuberculiform and clearly shifted forward off the caudal margin of the metatergum (the left horn also being nearly bifid), while the 1+1 central paramedian cones in front are a little higher than the lateral ones (Fig. 1A). All this is definitely evidence of the variability being purely individual.

The NHMW series of “*Diaphorodesmus attemsii*” syntypes, which contains 1 ♂ and 1 ♀ from Bibundi, 2 ♀♀ from Victoria, and a microscopic slide with the gonopods of a ♂ from Mukonje Farm, shows the same somatic variations as noted above (N. Akkari, in litt.). Thus, metatergum 4 of the ♂ from Bibundi (Fig. 1B) has typical horns, both rather high, slightly curved caudad and placed quite close to the posterior margin, whereas the cones in front are 2+2, the paramedian pair being slightly larger than the lateral one.

Hoffman, in his unpublished archives, provided the following distinctions between *Diaphorodesmus dorsicornis* from *Diaphorodesmus attemsii*, based solely on gonopod structure. The gonopod of “*Diaphorodesmus attemsii*” was drawn from a ♂ taken at Victoria, southwestern Cameroon (apparently, the Hamburg Museum collection, see Weidner 1960).

**Table:** 

*Diaphorodesmus dorsicornis* Gonopod postfemoral process (**pfp**) long and slender, apically curved and pointed, expanded distally from a broad base; an inconspicuous rounded lobule (**lo**) between base of **pfp** and solenomere lobe (**slo**) (Fig. 3A, B).	*Diaphorodesmus attemsii* Gonopod postfemoral process (**pfp**) relatively short, truncated apically, tapering regularly from a narrow base; a larger rounded lobe (**lo**) between base of **pfp** and solenomere lobe (**slo**) (Figs 3C, D & 5).

Hoffman used Verhoeff’s (1938) account of somatic differences (which actually do not hold, as the ZSM ♂ has the typical 2+2 cones in front of the dorsal horns!) to distinguish both *Diaphorodesmus dorsicornis* and *Diaphorodesmus attemsii* from what Hoffman evidently intended to describe as a new species. He also made several drawings of somatic and gonopodal characters, using a ♂ from Port Harcourt, southeastern Nigeria (Fig. 2). Its metatergum 4 may indeed show 3+3 cones in front of the horns (Fig. 2A), while its gonopod traits (Fig. 2D–F) match very closely those presented by Verhoeff (1938) for the ZSM ♂ (Fig. 4).

Comparing the gonopods of *Diaphorodesmus* samples from a number of often disparate localities across western Africa (see Porat 1894; Carl 1905; Attems 1931; Verhoeff 1938; VandenSpiegel et al. 2015, as well as our Figs 2D–F, 3–7), the variations observed in the relative sizes and shapes of **pfp**, **slo**, **lo** and **sph**, just like those of the above somatic features, seem to be random and too minor to consider more than individual. Therefore, we do not hesitate to formally synonymize *Diaphorodesmus attemsii* Verhoeff, 1938 with *Diaphorodesmus dorsicornis* (Porat, 1894), syn. n., treating the genus monospecific, albeit quite polymorphic. This conclusion is in accord with the vast distribution of *Diaphorodesmus dorsicornis* in southeastern Nigeria, southwestern Cameroon and Equatorial Guinea, western Africa (Fig. 12).

#### Diaphorodesmoides gen. n.

Taxon classificationAnimaliaPolydesmidaChelodesmidae

http://zoobank.org/A83F453D-5CA1-4EDA-840C-23DF5ABEFD45

##### Type species.

*Diaphorodesmoides lamottei* sp. n., by present designation.

##### Name.

To emphasize the strong resemblance to *Diaphorodesmus* Silvestri, 1896, particularly in sharing the conspicuous dorsal horns on metaterga 2–4.

##### Diagnosis.

A genus of Prepodesminae, Chelodesmidae that differs by the presence of a single, conspicuous, increasingly long, dorsomedian horn on each of metaterga 2–4, coupled with the ozopores not being borne on porosteles, but opening flush dorsolaterally on the surface of poriferous paraterga; the spiracles tubiform, unusually long and slender; and the gonopod telopodites being suberect, *in situ* directed forward, held parallel to each other, not crossing mesally; prefemoral (= densely setose) part erect, taking up ca 2/3 of total gonotelopodite length, without femorite, but with a more complex dorsal postfemoral process (**pfp**), set off from acropodite by a distinct cingulum; acropodite clearly twisted, divided parabasally into three large lobes, the middle of which forming a large solenomere lobe (**slo**) with only a minor solenomere proper (**sl**) on top, **slo** being neatly squeezed between a larger mesal uncus (**u**) and a smaller lateral branch (**lb**), both **u** and **lb** forming a solenophore.

#### Diaphorodesmoides lamottei sp. n.

Taxon classificationAnimaliaPolydesmidaChelodesmidae

http://zoobank.org/D6F84270-C6BE-4292-9BCB-EA715386AFA3

**Figure 8.**
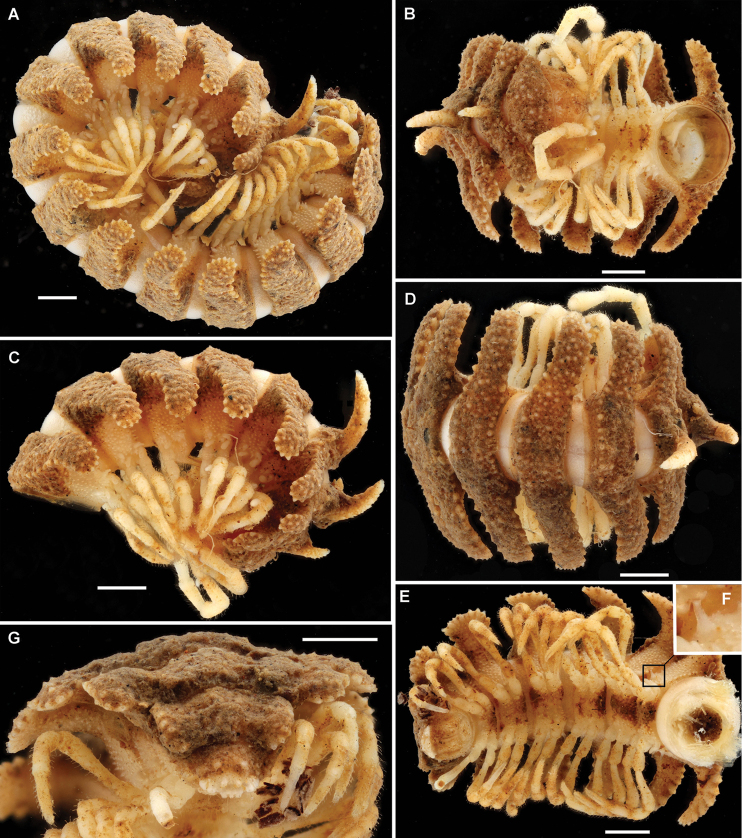
*Diaphorodesmoides lamottei* sp. n., ♂ holotype. **A** Habitus, lateral view **B–D** Anterior part of body, ventral, lateral and dorsal views, respectively **E** Caudal part of body, ventral view **F** Spiracle, subventral view **G** Last few body segments, caudal view. Scale bars: 1.0 mm (**A–E**, **G**), not to scale (**F**). Photos by J. Brecko.

**Figure 9.**
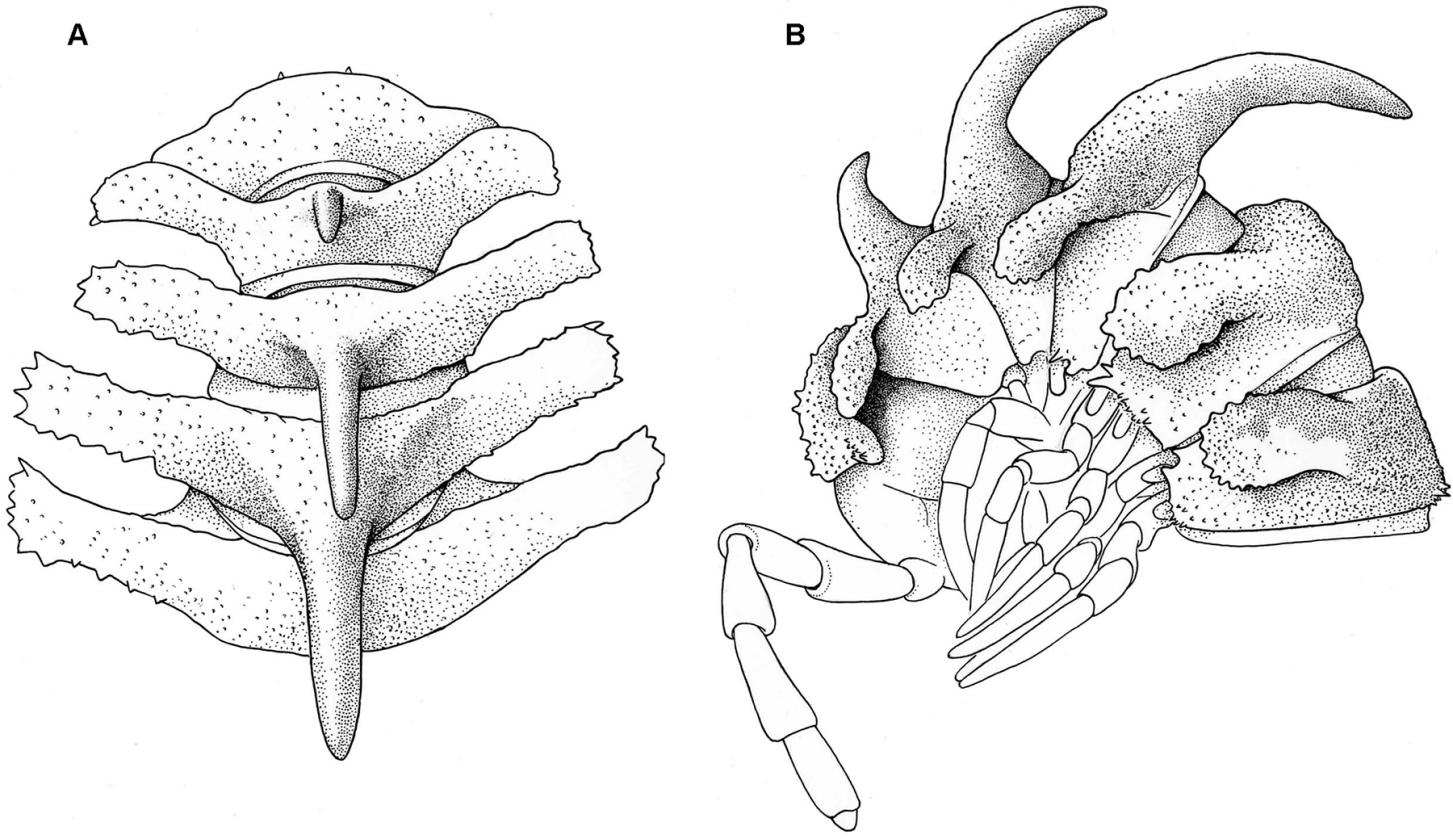
*Diaphorodesmoides lamottei* sp. n., ♂ paratype. **A, B** Anterior part of body, dorsal and lateral views, respectively. Del. N. Bertoncini (MHNH), drawn not to scale.

**Figure 10.**
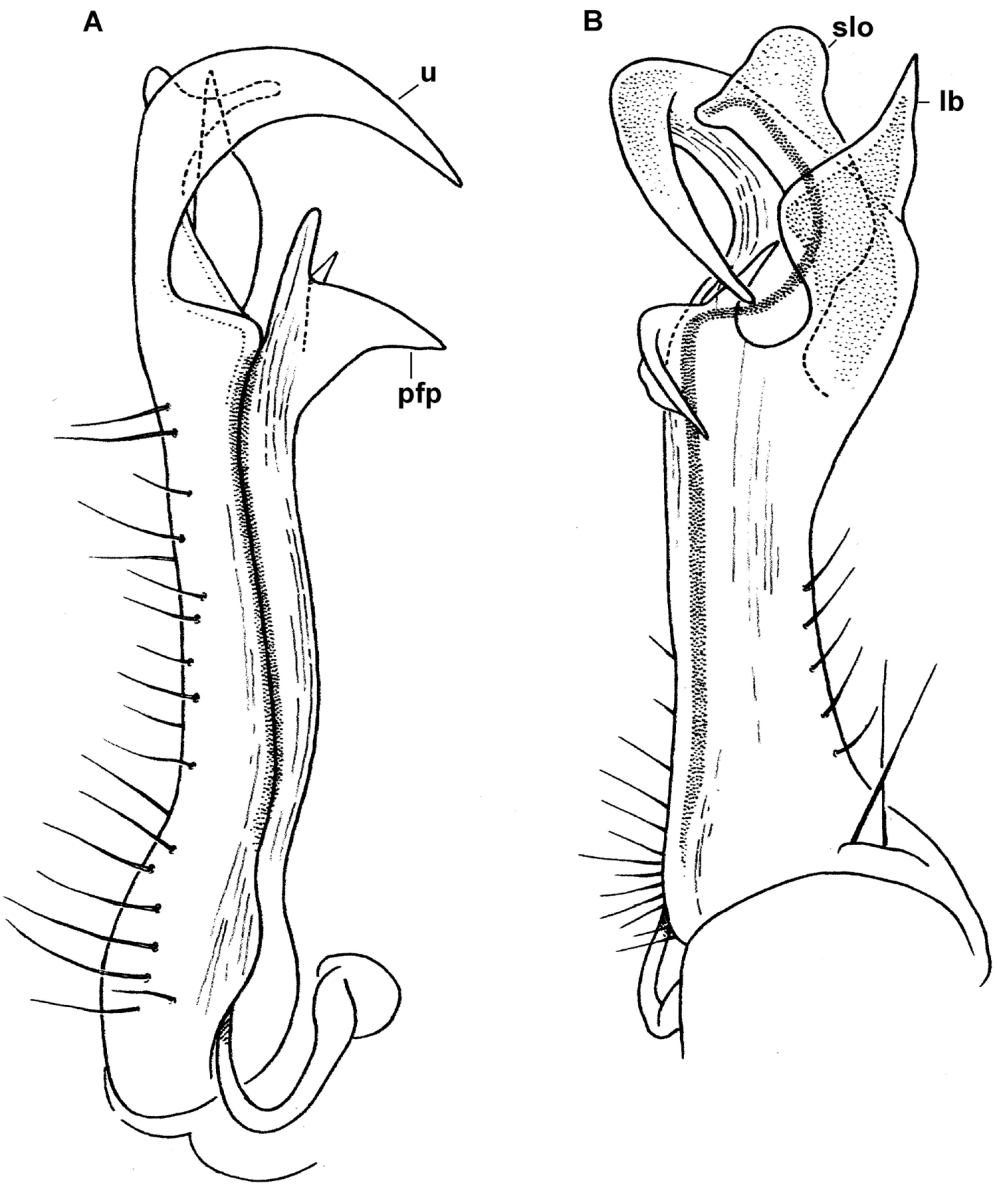
*Diaphorodesmoides lamottei* sp. n., ♂ paratype. **A, B** Right gonopod, mesal and sublateral views, respectively. Del. N. Bertoncini (MHNH), drawn not to scale.

**Figure 11.**
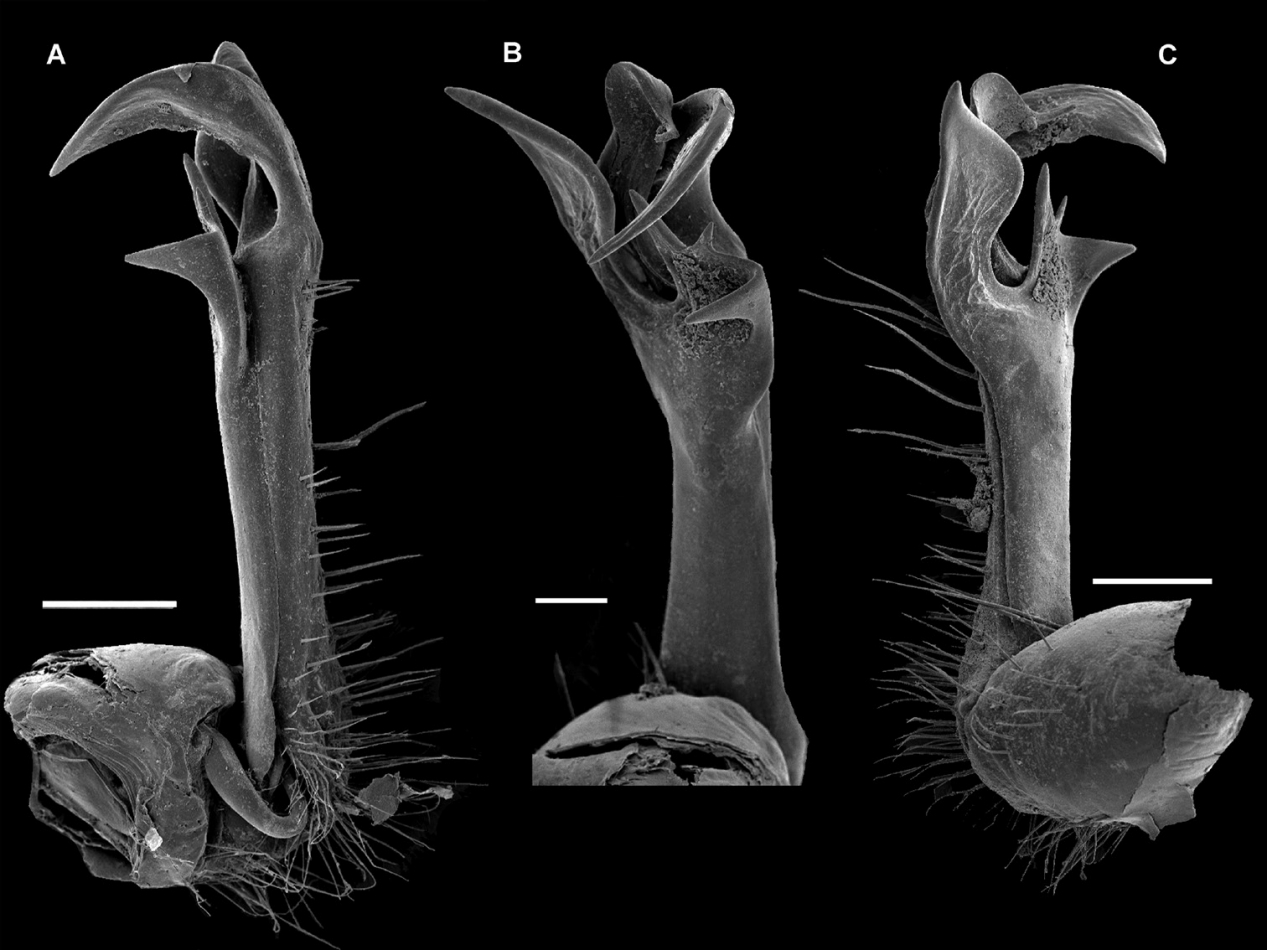
*Diaphorodesmoides lamottei* sp. n., ♂ holotype. **A–C** SEM micrographs of left gonopod, mesal, anterior and lateral views, respectively. Scale bars: 0.2 mm.

**Figure 12.**
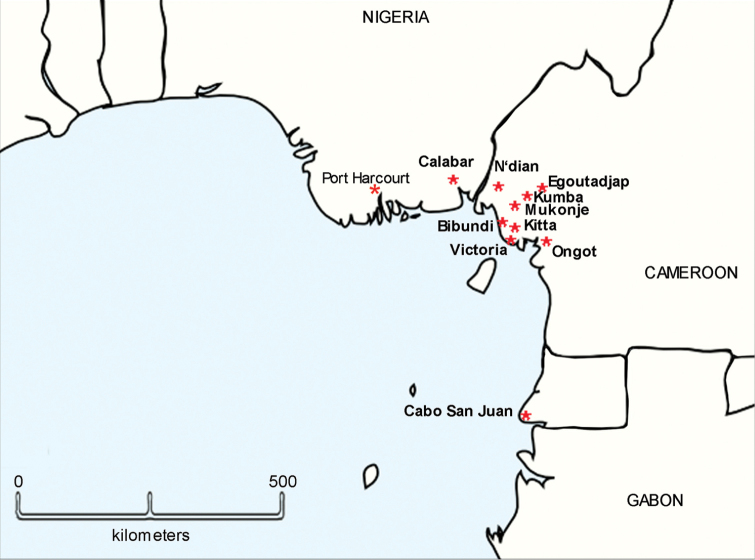
Distributions of *Diaphorodesmus dorsicornis* (Porat, 1894) (only known localities, arranged more or less from northwest to south; SE Nigeria: Port Harcourt; Osomba 56 mi from Calabar; SW Cameroon: N’dian, Egoutadjap, Kumba, Mukonje, Bibundi, Kitta, Victoria, Ongot; Equatorial Guinea: Cabo San Juan) and *Diaphorodesmoides lamottei* sp. n. (only Kumba).

Figs 8 , 9 , 10 , 11 , 12

##### Name.

To honour Maxime Lamotte, the collector.

##### Material examined.

**Holotype.** Cameroon: ♂ (MNHN JB253), KumbaEtam, 25.XI.1975, leg. M. Lamotte.

**Paratype.** Cameroon: 1 ♂ (MNHN JB253), same place, together with holotype.

##### Description.

Length of holotype ca 26 mm, width of midbody pro- and metazonae 2.0 and 5.7 mm, respectively. The sole ♂ paratype is ca 27 mm long, 2.1 and 5.8 mm wide on pro- and metazonae, respectively. Metaterga and epiproct dirty brown dorsally, with lighter granulations and tubercles (Fig. 8); head and ventral sides of paraterga a little lighter, brownish; antennae, sides, venter and legs light, yellowish.

Head densely granulate-microtuberculate and setose on dorsal face, interantennal isthmus about half as broad as diameter of antennal socket. Antennae long and only slightly clavate, *in situ* reaching behind body segment 3 when stretched dorsally; antennomeres 5 and 6 each with a dorso-apical group of tiny bacilliform sensilla; in length, antennomere 6>2=5>1>7; apical segment with usual four sensory cones.

Body with 20 segments (♂). In width, segment head < collum < segment 2 < 3 < 4 < 5 < 6 = 15; body rapidly tapering from segment 18 towards telson. Collum transversely ellipsoid, not covering the head from above; sides narrowly rounded; dorsal surface densely irregularly granulate-tuberculate (Figs 8C, 9B). Dorsum strongly and mostly regularly convex (Figs 8, 9). Only prozonae smooth and shining; metazonae dull, densely tuberculate-granulate all over, devoid of a cerategument, but in places clothed with a crust of earth dirt; dorsal surface of metaterga and ventral sides of paraterga with 6–8 irregular transverse rows of small grains, tubercles or short spines, only marginal rows being regular and, on paraterga, composed of ca 10 tubercles in each fore and caudal row, and of 5–6 at lateral edge; stricture smooth. Metaterga 2–4 each with an increasingly prominent, caudally curved and nearly sharp, microgranulate, subcylindrical, central horn (Figs 8A–D, 9). Metaterga 2–5 each with a small, but evident impression at base of paraterga, following paraterga (nearly) regularly convex, continuing the convex outline of mid-dorsal region. Paraterga very broad, set at about upper 1/3 of body, tips regularly rounded, mostly lying at about half of body height and slightly bent down; only paraterga 16–19 increasingly clearly drawn behind rear tergal margin, 19^th^ sharp. Sides below paraterga densely granulate, grains in caudal row being longer, spiniform and sharp. Ozopores barely visible, open flush on surface near midlength slightly above lateral edge of paraterga; pore formula untraceable. A thin, dark, axial line sometimes traceable through a transparent tegument, best visible on collum and prozonae. Pleurosternal carinae wanting. Limbus entire, translucent. Epiproct short, small, spade-shaped, strongly flattened dorsoventrally, subtruncate, dorsally granulate-tuberculate (Fig. 8G). Hypoproct densely granulate-tuberculate, roundly subtrapeziform, with 1+1 caudal setae very distinctly separated and borne on minute knobs (Fig. 8E). Paraprocts likewise densely granulate-tuberculate (Fig. 8E).

Sterna broad, nearly twice as broad as coxa length, almost flat, densely setose (Fig. 8E). Gonapophyses on ♂ coxae 2 vestigial. Spiracles (Fig. 8A, C, F) tubiform, remarkably long and slender. Legs very long, about 2.0 times as long as midbody height (♂), very slender; in length, femur > tarsus > tibia > prefemur = postfemur = coxa; claw very small, very slightly curved; ventral surface of tarsi densely setose, but forming no brushes.

Gonopod aperture transversely ovoid, large, its lateral and posterior edges slightly elevated, fully concealing gonocoxae and bases of telopodites. Gonopods relatively complex (Figs 10, 11). Coxites medium-sized, subcylindrical, fused at base to a small membranous sternal remnant, poorly setose distodorsally, including a pair of very closely placed, distalmost and particularly long setae. Cannulae slender, without peculiarities. Telopodites *in situ* directed forward, held subparallel to each other, suberect, not crossing each other mesally. Prefemoral (= densely setose) part erect, taking up ca 2/3 of total gonotelopodite length, without femorite, but with a relatively short, complex, tridentate, dorsal postfemoral process (**pfp**), set off from acropodite by a distinct cingulum; acropodite clearly twisted, divided parabasally into three large lobes, the middle of which forming a large solenomere lobe (**slo**) with only an indistinct, small solenomere proper on top, **slo** being neatly squeezed between a larger mesal uncus (**u**) and a smaller, subtriangular, lateral branch (**lb**), both **u** and **lb** forming a solenophore.

##### Remark.

At least at Kumba, the above new genus and species seems to occur sympatrically with *Diaphorodesmus dorsicornis* (Fig. 12). The label reading “KumbaEtam” is somewhat dubious. ‘Etam’ is a locality about 15 km NE of Kumba in Cameroon. The locality may therefore mean ‘between Kumba and Etam’ or ‘in the Kumba-Etam area’.

## Supplementary Material

XML Treatment for Diaphorodesmus

XML Treatment for Diaphorodesmus dorsicornis

XML Treatment for Diaphorodesmoides

XML Treatment for Diaphorodesmoides lamottei
